# Supplementary material for: Comparative Morphological, Ultrastructural, and Molecular Studies of Four Cicadinae Species Using Exuvial Legs
Source: Insects. 2019 Jul 6;10(7):199. doi: 10.3390/insects10070199 (PMC6681316; doi:10.3390/insects10070199)
Supplement: Supplementary file 1 [file insects-10-00199-s001.pdf]

Table S1. Exuvium information of specimen, collection locality and data, with accession number for each sample in this molecular study. Asterisked accession number are sequences obtained in this study.

Sequence_ID	Collection locality	Collection date	Voucher No	Accession No.
CA-1_CO1	Jijok-dong, Yuseong-gu, Daejeon, Korea	28-Jul-2018	2-19-0006-1	MK807119*
CA-2_CO1	Jijok-dong, Yuseong-gu, Daejeon, Korea	28-Jul-2018	2-19-0006-3	MK807120*
CA-3_CO1	Jijok-dong, Yuseong-gu, Daejeon, Korea	28-Jul-2018	2-19-0006-5	MK807121*
CA-4_CO1	Jijok-dong, Yuseong-gu, Daejeon, Korea	28-Jul-2018	2-19-0006-6	MK807122*
CA-5_CO1	Jijok-dong, Yuseong-gu, Daejeon, Korea	28-Jul-2018	2-19-0006-7	MK807123*
CA-6_CO1	Jijok-dong, Yuseong-gu, Daejeon, Korea	28-Jul-2018	2-19-0006-9	MK807124*
CA-7_CO1	Jijok-dong, Yuseong-gu, Daejeon, Korea	28-Jul-2018	2-19-0006-10	MK807125*
CA-8_CO1	Jijok-dong, Yuseong-gu, Daejeon, Korea	28-Jul-2018	2-19-0006-11	MK807126*
CA-9_CO1	Jijok-dong, Yuseong-gu, Daejeon, Korea	28-Jul-2018	2-19-0006-12	MK807127*
CA-10_CO1	Jijok-dong, Yuseong-gu, Daejeon, Korea	28-Jul-2018	2-19-0006-13	MK807128*
CA-11_CO1	Jijok-dong, Yuseong-gu, Daejeon, Korea	28-Jul-2018	2-19-0006-14	MK807129*
CA-12_CO1	Jijok-dong, Yuseong-gu, Daejeon, Korea	28-Jul-2018	2-19-0006-15	MK807130*
CA-13_CO1	Jijok-dong, Yuseong-gu, Daejeon, Korea	28-Jul-2018	2-19-0006-16	MK807131*
CA-14_CO1	Jijok-dong, Yuseong-gu, Daejeon, Korea	28-Jul-2018	2-19-0006-17	MK807132*
C. atrata	-	-	-	MG737717
HM-1-CO1	Cheolsan-dong, Gwangmyeong-si, Gyeonggi, Korea	27-Jul-2018	2-19-0007-1	MK807133*
HM-2-CO1	Cheolsan-dong, Gwangmyeong-si, Gyeonggi, Korea	27-Jul-2018	2-19-0007-2	MK807134*
HM-3-CO1	Cheolsan-dong, Gwangmyeong-si, Gyeonggi, Korea	27-Jul-2018	2-19-0007-3	MK807135*
HM-4-CO1	Cheolsan-dong, Gwangmyeong-si, Gyeonggi, Korea	27-Jul-2018	2-19-0007-5	MK807136*
HM-5-CO1	Cheolsan-dong, Gwangmyeong-si, Gyeonggi, Korea	27-Jul-2018	2-19-0007-6	MK807137*
HM-6-CO1	Cheolsan-dong, Gwangmyeong-si, Gyeonggi, Korea	27-Jul-2018	2-19-0007-7	MK807138*

HM-7-CO1	Cheolsan-dong, Gwangmyeong-si, Gyeonggi, Korea	27-Jul-2018	2-19-0007-8	MK807139*
HM-8-CO1	Cheolsan-dong, Gwangmyeong-si, Gyeonggi, Korea	27-Jul-2018	2-19-0007-9	MK807140*
HM-9-CO1	Cheolsan-dong, Gwangmyeong-si, Gyeonggi, Korea	27-Jul-2018	2-19-0007-10	MK807141*
HM-10-CO1	Cheolsan-dong, Gwangmyeong-si, Gyeonggi, Korea	27-Jul-2018	2-19-0007-12	MK807142*
HM-11-CO1	Cheolsan-dong, Gwangmyeong-si, Gyeonggi, Korea	27-Jul-2018	2-19-0007-13	MK807143*
HM-12-CO1	Cheolsan-dong, Gwangmyeong-si, Gyeonggi, Korea	27-Jul-2018	2-19-0007-14	MK807144*
HM-13-CO1	Cheolsan-dong, Gwangmyeong-si, Gyeonggi, Korea	27-Jul-2018	2-19-0007-15	MK807145*
HM-14-CO1	Cheolsan-dong, Gwangmyeong-si, Gyeonggi, Korea	27-Jul-2018	2-19-0007-16	MK807146*
HM-15-CO1	Cheolsan-dong, Gwangmyeong-si, Gyeonggi, Korea	27-Jul-2018	2-19-0007-17	MK807147*
HM-16-CO1	Cheolsan-dong, Gwangmyeong-si, Gyeonggi, Korea	27-Jul-2018	2-19-0007-18	MK807148*
HM-17-CO1	Cheolsan-dong, Gwangmyeong-si, Gyeonggi, Korea	27-Jul-2018	2-19-0007-19	MK807149*
H. maculaticollis	-	-	-	KY860344
MO-1-CO1	Ganghwa-eup, Ganghwa-gun, Incheon, Korea	31-Jul-2018	MBC_KIOM-2018-MO-1	MK807159*
MO-2-CO1	Ganghwa-eup, Ganghwa-gun, Incheon, Korea	31-Jul-2018	MBC_KIOM-2018-MO-2	MK807160*
MO-3-CO1	Jijok-dong, Yuseong-gu, Daejeon, Korea	18-Jul-2018	2-19-0131-1	MK807161*
MO-4-CO1	Jijok-dong, Yuseong-gu, Daejeon, Korea	18-Jul-2018	2-19-0131-2	MK807162*
MO-5-CO1	Ganghwa-eup, Ganghwa-gun, Incheon, Korea	31-Jul-2018	MBC_KIOM-2018-MO-3	MK807163*
MO-6-CO1	Ganghwa-eup, Ganghwa-gun, Incheon, Korea	31-Jul-2018	MBC_KIOM-2018-MO-4	MK807164*
MO-7-CO1	Jijok-dong, Yuseong-gu, Daejeon, Korea	18-Jul-2018	2-19-0131-3	MK807165*
MO-8-CO1	Jijok-dong, Yuseong-gu, Daejeon, Korea	18-Jul-2018	2-19-0131-4	MK807166*
M. opalifera	-	-	-	GQ527088
PK-1-CO1	Yeongdeungpo-dong, Seoul, Korea	29-Jul-2018	MBC_KIOM-2018-PK-2	MK807150*
PK-2-CO1	Jeongeup, Jeonbuk, Korea	31-Jul-2018	2-19-0130-1	MK807151*
PK-3-CO1	Jeongeup, Jeonbuk, Korea	31-Jul-2018	2-19-0130-2	MK807152*
PK-4-CO1	Yeongdeungpo-dong, Seoul, Korea	29-Jul-2018	MBC_KIOM-2018-PK-6	MK807153*

PK-5-CO1	Yeongdeungpo-dong, Seoul, Korea	29-Jul-2018	MBC_KIOM-2018-PK-8	MK807154*
PK-6-CO1	Yeongdeungpo-dong, Seoul, Korea	29-Jul-2018	MBC_KIOM-2018-PK-9	MK807155*
PK-7-CO1	Yeongdeungpo-dong, Seoul, Korea	29-Jul-2018	MBC_KIOM-2018-PK-10	MK807156*
PK-8-CO1	Yeongdeungpo-dong, Seoul, Korea	29-Jul-2018	MBC_KIOM-2018-PK-12	MK807157*
PK-9-CO1	Yeongdeungpo-dong, Seoul, Korea	29-Jul-2018	MBC_KIOM-2018-PK-14	MK807158*
P. kaempferi	-	-	-	MG737816
†*A. cardamines*	-	-	-	MH420365

†, outgroup; CA, *Cryptotympana atrata*; HM, *Hyalessa maculaticollis*; MO, *Meimuna opalifera*; PK, *Platypleura kaempferi*.
